# Theta band transcranial alternating current stimulations modulates network behavior of dorsal anterior cingulate cortex

**DOI:** 10.1038/s41598-017-03859-7

**Published:** 2017-06-15

**Authors:** Keiichi Onoda, Toshikazu Kawagoe, Haixia Zheng, Shuhei Yamaguchi

**Affiliations:** Department of Neurology, Shimane University, Shimane, Japan

## Abstract

Dorsal anterior cingulate cortex (dACC) is an important region in the processing of both cognition and affect. Recently, transcranial brain stimulation has been used to modulate cortical activity, but it is unclear whether this stimulation has a specific effect on dACC. Based on EEG evidence that frontal midline theta activity is generated in dACC, we hypothesized that transcranial alternating current stimulation (tACS) with theta band frequency would modulate neural networks including dACC. In this study, we examined the effects of theta band tACS on functional networks and emotional state. Graph theory analysis for resting-state functional MRI data revealed that theta band tACS decreased functional integration and hub capacity in dACC, and the attenuation of dACC network function was associated with emotional state change. Overall, these results demonstrate that theta band stimulation can modulate dACC.

## Introduction

Dorsal anterior cingulate cortex (dACC) plays a pivotal role in the processing of both cognition and affect^1, 2^, but specific roles of dACC have been proposed for decades. Furthermore, numerous imaging studies have reported abnormal dACC function in various neuropsychiatric disorders^3^. dACC was a target of psychosurgery for severe psychiatric disorders^3^ and is one recent target of deep brain stimulation^4^. If non-invasive brain stimulation is able to modulate dACC, this would pave the way for further examinations of dACC function as well as provide safer interventions for psychiatric disorders. However, it remains unknown whether non-invasive brain stimulation can modulate a specific region.

Recently, brain stimulations, including transcranial alternating current stimulation (tACS) and transcranial magnetic stimulation (TMS), have been increasingly explored for their utility in investigating the effect of cortical modulation on various neural networks. In particular, tACS offers a useful way to emulate naturally occurring rhythms in the cortex^5^. For instance, 20 Hz tACS decreased TMS-induced phosphene threshold (increased excitability of visual cortex)^6^, which suggests that tACS can be used as a tool for establishing a causal link between rhythmic cortical activities and their functions. However, such frequency-dependent neural modulations have only been reported for cortex beneath the cranium. To date, there has been no evidence for similar effects on regions far from the scalp. Theta frequency rhythmic activity is observed in the mid-frontal area, which researchers have dubbed frontal midline theta (FMT). The broader literature suggests that FMT emergence is associated with cognitive effort, working memory, and even anxious temperament, and that dACC generates this oscillatory activity^7^. Prior magnetoencephalography (MEG) results revealed that FMT power was attenuated during theta band tACS^8^. The cumulative evidence for FMT suggests the attractive hypothesis that theta band tACS would modulate dACC function.

Here, we investigated the effects of theta band tACS on a resting state functional network, as measured via resting state functional magnetic resonance imaging (rsfMRI). Graph theory provides a framework in which the topology of complex networks can provide comprehensive information about the local and global organization of brain circuitry. Applying graph theory to rsfMRI data can help quantify various network properties in each region, including functional segregation, integration, and hub^9^. In this study, we hypothesized that theta band tACS would modulate dACC graph properties.

## Results

**Figure 1:**
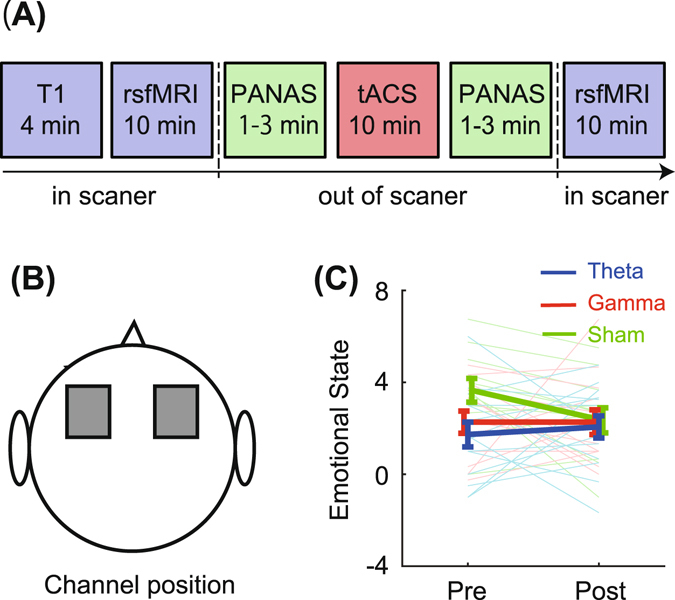
(**A**) Experimental procedure. Measurements of resting-state fMRI and emotional state used by Positive and Negative Affect Schedule (PANAS) were conducted before and after tACS session. (**B**) Stimulation electrodes were placed at bilateral frontal areas (F3 and F4 in 10–20 system). (**C**) Emotional state change in each stimulation group. Higher values indicate positive mood. Thick and light thin lines show group averages and individual scores, respectively. Error bars denote standard error.

RsfMRI and emotional state were assessed before and after tACS (1.0 mA, 10 min) applied over the dorsolateral frontal site (Fig. 1A,B). Healthy young volunteers were randomly assigned to a theta band (6 Hz), gamma band (30 Hz: active control), or sham stimulation group. First, participants of the theta band tACS group did not show any shift of self-rated emotional state between pre- and post-stimulation (Fig. 1C).

**Figure 2:**
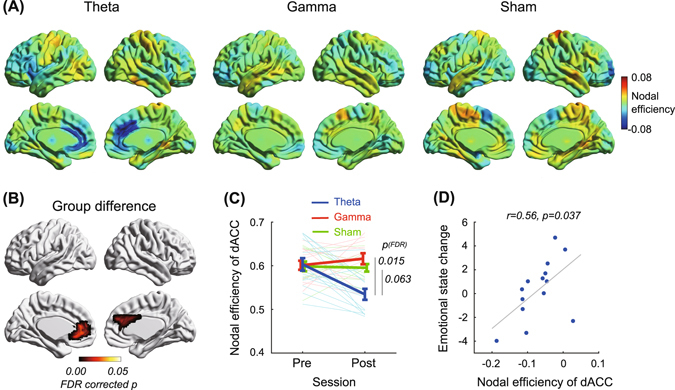
Effects of tACS on nodal efficiency. (**A**) Bilateral and medial views of nodal efficiency changes (represented by average of network density 0.2–0.3) in theta band, gamma band, and sham tACS groups. Hot and cool colors denote increase or decrease of nodal efficiency, respectively. (**B**) Significant group difference of nodal efficiency. FDR-corrected p-values were obtained by a permutation test (iteration: 5000), and multiple comparison corrections were applied at the node level. (**C**) Nodal efficiency changes in dorsal anterior cingulate cortex (dACC) are shown in average (thick line) and individual (light thin line) levels. Error bars denote standard error. (**D**) Relationship between nodal efficiency change of dACC and emotional state change.

Second, we used graph theory to analyze topological properties of the resting-state functional network for all nodes (See methods, Supplementary Figs S1 and S2). The analysis revealed unique effects of theta band stimulation on dACC. Theta band stimulation notably decreased nodal efficiency in dACC as well as rostral regions (*ps*_*bootstrap*_ < 0.001, Fig. 2A), which was defined as the average inverse of shortest path length^10^ and is thought to reflect functional network integration, whereas gamma band and sham stimulation did not cause significant changes in dACC nodal efficiency. A group comparison revealed a significant main effect of group (FDR-corrected *p*_*permutation*_ = 0.015, Fig. 2B), such that the nodal efficiency change of dACC for theta band tACS decreased more than for the other two groups (FDR-corrected *ps*_*permutation*_ < 0.063, Fig. 2C) and was similar in the rostral portion. On the other hand, tACS had no significant effect on nodal efficiency of all other nodes, including the dorsolateral prefrontal cortex under stimulation channels. Importantly, a correlational analysis confirmed that nodal efficiency change of dACC was positively correlated with emotional state change in the theta band tACS group (*r* = 0.56, *p* = 0.037, Fig. 2D). The other two groups showed no correlations between the measures (*rs* < 0.37, *ps* > 0.23).

**Figure 3:**
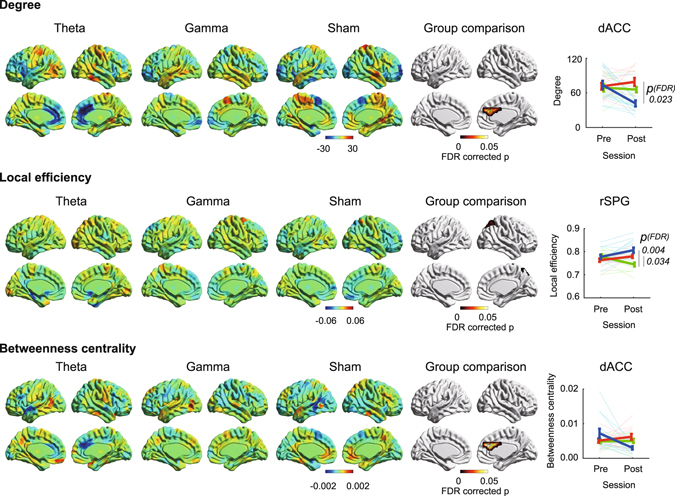
Effects of tACS on degree (top), local efficiency (middle), and betweenness centrality (bottom) in theta band, gamma band, and sham groups. Hot and cool colors denote increase or decrease of graph measures, respectively. Each figure consists of bilateral and medial views. FDR-corrected permutation test was used for group comparisons (iteration: 5000), and multiple comparison corrections were applied at the node level. Figures at the far right show graph measure changes in dorsal anterior cingulate cortex (dACC) or right superior parietal gyrus (rSPG). Thick and light thin lines show group averages and individual scores, respectively. Error bars denotes standard error. Blue, red, and green denote theta band, gamma band, and sham group.

As was the case for nodal efficiency, degree and betweenness centrality, reflecting functionality as a hub^11^, decreased in dACC after theta band stimulation (*ps*_*bootstrap*_ < 0.001, Fig. 3) compared to sham stimulation (FDR-corrected *ps*_*permutation*_ < 0.05, Fig. 3). The other nodes did not show any significant effects on these measures. These unique effects on dACC were replicated when applying the absolute threshold method (Supplementary Fig. S3). However, local efficiency, which reflects functional segregation^10^, showed no robust changes due to theta band stimulation. In contrast, a significant main effect of group on local efficiency was observed in the right superior parietal gyrus (FDR-corrected *p*_*permutation*_ = 0.008, Fig. 3), in which local efficiency was decreased in the region after sham stimulation (*ps*_*bootstrap*_ < 0.001) compared to other stimulations (FDR corrected *ps*_*permutation*_ < 0.034, Fig. 3).

**Figure 4:**
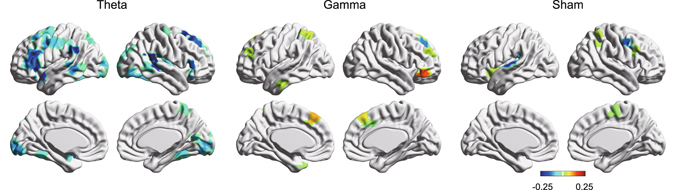
Efficiency between right dorsal anterior cingulate cortex and others in each group. Colored regions (hot and cool) show significant increased or decreased efficiency (bootstrap p < 0.001).

When examining relationships between the dACC and other regions, we found a significant reduction in efficiency between the dACC and other whole brain regions (including the lateral prefrontal cortex and amygdala) for the theta band tACS group (*ps*_*bootstrap*_ < 0.001), but such a pattern was not obtained for the other groups (Fig. 4).

To corroborate the evidence of graph theory analysis, we conducted a region-of-interest (ROI)-based analysis and independent component analysis (ICA). In ROI-to-ROI level, theta and gamma band stimulation evoked similar patterns of functional connectivity (Supplementary Fig. S4). In both groups, the anterior part of the brain had decreased connectivity with the central area, while the posterior part had increased connectivity. Importantly, dACC and dorsolateral prefrontal cortex did not show notable modulation and there was no significant group difference. Therefore, modulations in ROI-to-ROI connectivity level were considered secondary effects of tACS, and ROI-to-ROI analysis might not fit to detect the effects of tACS. We also performed ICA to divide rsfMRI data into functional networks including salience networks, which consisted of the ACC and anterior insula. The functional connectivity between the salience network and other networks (executive control, auditory, and visual networks) was decreased in theta band tACS group (FDR-corrected *ps* < 0.05, Supplementary Fig. S5). However, inter-component connectivity did not reveal a significant group difference.

Additionally, we examined the effects on basic signal changes using blood oxygenation level dependent (BOLD) signal and the amplitude of low frequency fluctuations (ALFF) (Supplementary Fig. S6). BOLD signal changes in each group exhibited no regional difference and showed no significant group difference. ALFF is the total power of low frequency oscillation (0.01–0.10 Hz), which reflects neural activity^12^. Like BOLD signal, ALFF did not show any significant group difference. These results suggest that network modulation by tACS depends on connectivity pattern rather than basic signal changes.

## Discussion

As predicted, our study demonstrated that theta band tACS served to modulate dACC network properties, whereas gamma band and sham tACS exhibited no notable effects. Thus, theta band tACS has a frequency-specific impact on dACC. Degree, nodal efficiency, and betweenness centrality for dACC were decreased by theta band tACS, suggesting that topological distances between dACC and other regions increased and that the hub function of dACC declined after the stimulation.

How could theta band tACS modulate network activity of dACC? First, the effect of electrical stimulation on dACC should be considered. There is concern that stimulation on the bilateral prefrontal areas (F3/F4) might not be able to affect brain activity because of the proximity of stimulus electrodes. However, some studies revealed that stimulation over these regions improved performance of executive functions, such as inhibition^13^ and shifting^14^. These behavioral studies suggest that stimulation on the F3/F4 montage could affect at least some brain functions. A simulation study revealed that current density underneath stimulus electrodes was localized and that cortex far from stimulus electrodes showed high current density^15^. Anatomically, dACC is reciprocally projected to dorsolateral prefrontal cortex^16^. Accordingly, tACS in the present study could directly and/or indirectly impact dACC. Then, why does only theta band tACS modulate functional connectivity or the dACC network? Rhythmic excitability has been proposed to instantiate transient functional networks between spatially distant regions^17^. Many EEG and MEG studies suggest that theta rhythm oscillation in the brain is likely to have multiple generators, especially in the frontal lobe and dACC^18^. dACC is connected to a number of different brain regions^19^, and theta activity of front-central areas shows a phase-lead relative to activity of other brain regions^20^. These findings suggest that FMT generated in the dACC contributes to the integration of distant theta activity of other brain regions. FMT appears in discrete bursts that last a few seconds (range from 1 to >10 s), and there is no time-locked relationship between task initiation and the appearance of FMT^21^. tACS induces up- and down-regulation of the firing rate in an oscillatory manner without changing the average firing rate over a long time period^22^. In this study, the phase of spontaneous FMT did not coincide with the phase of theta band tACS in the resting state. This discrepancy might cause impairment of the integration function in dACC, resulting in decreased network function of the region after stimulation. This discussion implicitly assumes the presence of direct effects of tACS on dACC. In line with this notion, it is reported that theta band tACS attenuated FMT power^8, 23^.

A recent study of Cabral-Calderin *et al*., which examined the effects of alpha and gamma band tACS (Cz/Oz and P5/P6) on resting-state networks, also reported frequency-dependent manner^24^. Their finding was that alpha band tACS increased functional connectivity while gamma band tACS decreased it. Cabral-Calderin *et al*. explained that the antagonism between the frequency bands reflects their functional differences. In general, alpha power is more related to the functional inhibition of task-irrelevant regions^25^, whereas gamma power is associated with to BOLD activity^26^. Their results might be explained by opposite modulations of alpha power, in which alpha band tACS increased alpha power while gamma band tACS decreased alpha power via cross-frequency coupling^27^. The report of Cabral-Calderin *et al*. is seemingly not corresponding with our finding. Alpha and theta bands have the common feature of a synchronization phenomenon between long-distance regions^28^, but the functional aspects of alpha and theta band activities are different. FMT naturally entrains activities in disparate neural systems, resulting in cross-cortical information transmission^7^. Thus, increased FMT power suggests an enhancement in the functional activity of dACC, and this manner is opposite to that of alpha band activity. The phase discrepancy between spontaneous EEG and tACS could be critical in the case of the theta band; however, alpha band tACS might not cause such a discrepancy because such activity synchronizes the stimulation^27^. In the case of the gamma band, phase discrepancy would not appear because the activity is a local phenomenon. Gamma band tACS might independently strengthen neural activity in each local region, resulting in an attenuated long-distance functional connection. However, we did not find any notable effects on functional connectivity between the dorsolateral prefrontal cortex and the other regions. A suitable frequency of gamma band tACS might be higher than that of our study (30 Hz) because Cabral-Calderin *et al*. found effects of 40 Hz tACS^24^.

It is unknown if non-invasive brain stimulations are able to selectively modulate regions far from scalp. A few studies reported stimulus-induced network changes (including the subcortex) during rest. However, such alterations occurred in multiple cortex-subcortex circuits^29, 30^. This suggests that non-invasive brain stimulation has broad rather than region-specific effects on the brain^15^. Another study suggested that transcranial direct current stimulation of the prefrontal area remotely activated the interconnected midbrain during a face evaluation task^31^, but different regions might be active if another task is used. Region-specificity of stimulus effects should be considered more carefully in future studies. We demonstrated a unique effect of theta band tACS on the dACC during rest, but a variety of behavioral studies combined with tACS-fMRI measures are necessary to reinforce our findings.

Behavioral effects of theta band tACS have already been reported in some studies, but the effects are contradictory. For instance, midfrontal tACS slowed down response times during a spatial conflict task^32^, and theta band tACS during a working memory task disrupted the task performance associated with decreased FMT amplitude^8^. Phase mismatch between FMT and tACS might have caused performance deterioration. Conversely, in some reports, tACS improved visual working memory^33^, short term memory^34^, reversal learning^35^, and fluid intelligence performance^23^. These studies used different methodology, including electrode location, stimulus frequency (fixed or individualization based on EEG), stimulus intensity, and timing of the behavioral measure (during or after stimulation). There is no clear explanation of the incongruity thus far, and systematic studies would be warranted to solve the issue. At least, this evidence implies the capability of tACS for up- and down-regulation in task performance, which likely depends on task type.

Why was the decreased network measure associated with deteriorated mood in the theta band tACS group? A possible answer is an alteration in the evaluation/appraisal processing of emotion. The lateral prefrontal cortex (mainly left)^36, 37^ and dACC^38^ are involved in the evaluation/appraisal of emotion, and the amygdala^39^ is involved in emotion processing. Both the lateral prefrontal cortex and amygdala showed decreased efficiency with dACC after theta band tACS, resulting in altered processing of emotion evaluation/appraisal. This interpretation corresponds to the integration function of dACC^7^. On the other hand, theta band tACS might be useful for the treatment of depression. Numerous rsfMRI studies on depression have been reported^40^, and our group also suggested that depression was associated with increased network function of ACC^41^. The dorsal and rostral ACC in depression has shown increased functional connectivity with the prefrontal cortex, insula, amygdala, basal ganglia, and thalamus^42–46^. A dynamic causal modelling study revealed that effective connectivity from dorsal to rostral ACC was strengthened in depression^47^. If theta band tACS attenuates exacerbated network function of dACC, it might improve symptoms in depression. However, as there is a discrepancy between predictions from functional network change and subjective mood change, the cause for this discrepancy requires further investigation.

One limitation of the current study is a lack of systematic evaluation for subjective sensation during and after tACS. Kanai *et al*. reported that the visibility of phosphenes elicited by tACS depends on the frequency of stimulation^48^; however, the phosphenes observed by Kanai *et al*. were elicited on the level of retina and not in cortical structures^49^. In the current study, theta band tACS caused decreased efficiency between dACC and visual cortex. Phosphene-related eye movement and/or the control might be involved in dACC activity even though participants were instructed to gaze at a fixation point and there was no spontaneous report about vision. To verify that the theta band tACS effect was on ACC rather than the retina, quantitative evaluations of subjective sensation and concurrent electro-oculogram are warranted.

We discovered that theta band tACS is able to modulate dACC network function. Our evidence is important in two respects. First, this study contributes to the existing research on dACC function. Modulation of dACC function by theta band tACS allows us to investigate potential causal effects on various cognitive and affective processes associated with this region. Second, the present findings may be applied to our clinical understanding of psychiatric disease. dACC is a target of previous psychosurgery and recent deep brain stimulation for severe psychiatric disorders^3, 4^ and theta band tACS could be an useful alternative intervention.

## Methods

### Participants

We investigated 45 participants (Mean age 23.1 ± 2.9 years old; 31 women, 14 men) without any diagnosis of psychiatric or neurological illness. All participants gave their written informed consent to participate after listening to a detailed explanation about the study design and possible risks. The study was approved by the medical ethics committee of Shimane University, and brain stimulation was performed in accordance with the guidelines of Woods *et al*.^50^. Participants were randomly assigned to three groups: theta band (6 Hz); gamma band (30 Hz); and sham stimulation. They underwent resting-state fMRI, and subjective evaluations of their emotional state before and after the tACS session. Six participants were excluded from the analysis due to excessive head movement during resting state fMRI (over 3 mm). There were no significant differences in age or sex ratios between groups (theta band group: 22.9 ± 2.4 years old, 10 women, 4 men; gamma band group: 22.4 ± 1.7 years old, 7 women, 6 men; sham group: 23.4 ± 3.5 years old, 9 women, 3 men).

### Experimental procedure

After informed consent, participants were provided with a procedural overview of the experiment. Participants were told that the experiment’s purpose was to examine the effects of weak electrical stimulation on brain networks, and they were not able to know any parameters of tACS except the duration. After instructions about MRI measurements (see below), structural and functional images were acquired. Participants left the scanner once to complete emotional state evaluations and tACS, and were instructed to sit in a chair and rest during tACS. tACS was performed with parameters in the following section, and emotional state evaluations were performed before and after tACS. Next, measurement of functional data at post-tACS was performed.

### Transcranial Alternating Current Stimulation

A battery-driven stimulator (NeuroConn, Ilmenau, Germany) was used to deliver an alternating current to the scalp via two electrodes. Electrodes (5 × 7 cm) were placed over the F3 and F4 area according to the 10–20 EEG system and were fixed with two rubber bands. This montage was used in studies examining transcranial direct current stimulation effects on executive functions, which demonstrated improved behavioral task performances^13, 14^. A simulation study indicated that even regions far from the scalp between closely-positioned electrodes are expected to have higher current distribution^15^. The dorsolateral prefrontal cortex under F3/F4 is reciprocally linked with dACC^16^. Based on this evidence, we expected that tACS could affect activity of dACC. Impedance was kept below 5 kΩ. Stimulation frequencies were 6 Hz for the theta band, 30 Hz for the gamma band, or no stimulation for the sham group. Stimulation current and duration were 1.0 mA (peak-to-peak) and 10 min, respectively. Participants sat in a chair during tACS. We asked participants to freely report feelings and pain after tACS and post rsfMRI session. No one reported excessive pain or discomfort.

### Emotional state evaluation

The Positive and Negative Affect Schedule Scale (PANAS^51^ Japanese version^52^) contains positive (e.g., interested, proud) and negative (e.g., ashamed, irritable) mood descriptors, with each item rated on a scale from 1–6: 1 (very slightly or not at all) to 6 (extremely). Participants completed the PANAS just before and after the tACS session. The scores were transferred by min-max normalization to correct inter-individual difference of deviation of scores, and the normalization process was applied to merged data including pre- and post-tACS at each individual level (standard deviation of individual, min: 0.2, max: 6.1 at 6 points scale). We calculated total positive and negative affective scores, respectively. After reverse scoring of negative items, positive and negative scores were summed to derive the total emotion score, such that higher scores indicated more positive mood. Differences between emotional states before and after tACS (Post - Pre) were calculated for each participant.

### MRI measurement

Imaging data were acquired using a Phillips 3.0 T scanner (INGENIA CX). Before the functional scans, T1-weighted images of the entire brain were measured (170 sagittal slices, slice thickness = 1.2 mm, repetition time = 6.8 msec, echo time = 3.1 msec, flip angle = 9°, matrix size = 256 × 256, field of view = 256 × 256 mm^2^, isotropic spatial resolution = 1 mm). For functional scans, forty axial slices parallel to the plane connecting the anterior and posterior commissures were measured using an echo planer imaging sequence (repetition time = 2500 msec, echo time = 30 msec, flip angle = 80°, scan order = ascending, matrix size = 64 × 64, field of view = 212 × 212 mm^2^, isotropic spatial resolution = 3.3 mm, slice thickness = 3.2 mm, gap = 0.8 mm, scans = 240). Measurements of resting-state fMRI lasted 10 min 10 seconds, including 4 initial dummy scans. Participants were instructed not to move their head in the scanner. In addition, participants were instructed to remain awake while looking at a fixation point and to think of nothing.

### fMRI preprocessing

SPM12 was used for preprocessing. Functional images were realigned to remove any artifacts from head movement and to correct for differences in image acquisition time between slices. Six participants who moved their head excessively (over 3 mm) were excluded from the following analysis. The remaining participants did not show any significant group differences (absolute max head movement, theta: 1.2 ± 0.7 mm, gamma: 1.1 ± 0.8 mm, sham: 0.7 ± 0.4 mm; framewise movement (individual median)^53^, theta: 0.08 ± 0.02 mm, gamma: 0.08 ± 0.02 mm, sham: 0.07 ± 0.02 mm). Next, the functional images were normalized to the standard space defined by a template T1-weighted image using the DARTEL method^54^ and resliced with a voxel size of 3 × 3 × 3 mm^3^. After spatial preprocessing, temporal preprocessing was performed using CONN toolbox. Effects of head movement parameters and BOLD signals from white matter and cerebrospinal fluids were removed at each voxel. Temporal smoothing was performed using the band-pass filter: 0.01–0.08 Hz.

### Parcellation

Grey-matter was parcellated into regions of interest using a k-means algorithm^55^. Each region of interest was required to serve as a distinct node in a graph, and the parcellation approach was developed to minimize the variation in nodal volume. The K-means algorithm with a squared Euclidean distance measure for clustering was applied to each region of an Automated Anatomical Labelling (AAL) template, and voxels of each region were divided into sub-regions. The number of clusters (k) for each AAL region was set to a rounded integer of Vi/(2 × Vmin), where Vi was the mean number of voxels included in bilateral areas of each AAL region and Vmin was the smallest number of voxels among all AAL cerebrum regions (corresponding to amygdala). This means that voxels in each AAL region were separated into k smaller ROIs based on just the spatial coordinates. Based on this procedure, 116 AAL regions were separated into 312 regions (see Supplementary Fig. S1).

### Connectivity Matrix

Mean time courses of the BOLD signal were calculated for voxels within each region and Pearson correlations for all time course pairs were computed for each participant. To construct a graph network, each functional connectivity matrix was converted to a binary network using a density threshold, which is equivalent to the ratio between the number of edges and all possible edges. The rationale for thresholding using network density was to focus on the topology of relatively sparse networks^56^ and to avoid spurious results yielded by direct comparisons of graph measures between networks with different numbers of connections^57^. The network density was set to a range from 0.05 to 0.50 (step: 0.01), and we applied graph theory analysis to all thresholded networks for each individual. When studying the topological properties of graphs across individuals, each node should be connected to the whole network via at least one neighbor for all subjects (i.e., degree > 0 for all nodes)^58^. Individual minimum network density to construct such graphs was 0.12 ± 0.05. Therefore, we computed averaged graph measures over the network density range 0.20–0.30 as representative values. Supplementary Fig. S2 shows the procedure of the computing graph measures. To check the robustness of our results in graph theory, data were reanalyzed using an absolute thresholding aproach^59^. Pearson correlation criteria were set to 0.80–0.40 (step: 0.01).

### Graph theory analysis

Node degree, nodal efficiency, local efficiency, and betweenness centrality of each node were computed as measures of functional integration, functional segregation, and centrality, respectively, using the Brain Connectivity Toolbox (BCT, sites.google.com/site/bctnet/). These measures were selected because they are free from connectedness restrictions^10^.

Node degree is defined as the number of links connected to that node, which is considered a basic and important measure.

Nodal efficiency is a measure of functional integration and is considered the average inverse shortest path length:

1 $${{\rm{E}}}_{i}=\frac{1}{n-1}\sum _{i,j\in N,j\ne i}{d}_{ij}^{-1}\,$$

where E_i_ is the efficiency of node i, n is the number of nodes, and d_ij_ is the shortest path length between nodes i and j. The path length represents the number of processing steps along the routes of information transfer among brain regions. Nodal efficiency measures the ability of information propagation between a given node i with the rest of the nodes in a network.

Local efficiency is a measure of functional segregation, which is the ability for specialized processing to occur within densely interconnected groups of brain regions^10^. We computed local efficiency as follows:

2 $${{\rm{E}}}_{loc,i}=\frac{1}{{k}_{i}({k}_{i}-1)}\sum _{j,h\in N,j\ne i}{a}_{ih}{a}_{ij}{[{d}_{hj}({N}_{i})]}^{-1}\,$$

where E_loc,i_ is the local efficiency of node i, ki is the degree of node i, a_ih_ is connection status (1 or 0) between i and h, and d_hj_(N_i_) is the length of the shortest path between h and j that contains only neighbors of i.

Betweenness centrality was calculated as follows:

3 $${{\rm{b}}}_{i}=\frac{1}{(n-1)(n-2)}\sum _{h,j\in N,h\ne i\ne j}\frac{{P}_{hj}(i)}{{P}_{hj}}\,$$

where P_ij_ is the number of shortest paths between h and j, and P_hj_(i) is the number of shortest paths between h and j that pass through i. Betweenness centrality of a node captures the influence of the node over the information flow between all the other nodes in the network. Higher betweenness centrality indicates that the region functions as a hub.

Because we found unique effects of theta band tACS on network measures of dACC (Figs 2 and 3), efficiency (reverse of shortest path length) between the dACC and others was estimated to investigate which connections were altered by the stimulations.

### Statistical analysis

For measures of each node in graph theory analysis, differences between pre- and post-tACS were calculated for each participant. Bootstrap tests were applied for the difference of each node for each group to detect significant change by the tACS. For the graph measure’s changes of nodes which showed significant change (p < 0.001) in any group at the test, permutation tests were performed to examine the effect of stimulation type and to control for false discovery rate (FDR) at region level^60^. We first computed the F values of ANOVA for all permuted data of the nodes, and selected the maximum values in each node. Accordingly, histogram distributions of the maximum F values across the nodes were obtained. Then, we calculated the p-value as the ratio between the number of patterns with greater maximum F value than observed data and the total number of permutations. In post-hoc analysis, the significance of the difference between any two groups was verified using same procedure. Iteration of the bootstrap and permutation tests was 5000. To illustrate regions that showed a significant effect of stimulation types, brain maps were created using BrainNet Viewer (www.nitrc.org/projects/bnv/).

### Additional analysis

Graph theory analysis revealed the notable effects of theta band tACS on network properties of dACC. To verify the validity of our evidence, we also performed ROI-to-ROI analysis and ICA for resting-state fMRI. Group ICA toolbox (GIFT, www.mialab.mrn.org/software/) was used for ICA. The analysis methods are described in each figure legend. In addition, to check basic signal change, BOLD signal and ALFF were calculated and compared to the effects of tACS between groups. ALFF was computed using functions of the resting-state fMRI data analysis toolkit (REST, www.restfmri.net/forum/REST_V1.8).

## Electronic supplementary material

Supplementary Information
