# Supplementary material for: Theta band transcranial alternating current stimulations modulates network behavior of dorsal anterior cingulate cortex
Source: Sci Rep. 2017 Jun 15;7:3607. doi: 10.1038/s41598-017-03859-7 (PMC5472616; doi:10.1038/s41598-017-03859-7)
Supplement: Supplementary file 1 — Supplementary Information [file 41598_2017_3859_MOESM1_ESM.pdf]

Supplementary Figures and Tables

Theta band transcranial alternating current stimulations modulates network behavior of dorsal anterior cingulate cortex.

Keiichi Onoda[1]*, Toshikazu. Kawagoe[1], Haixia Zheng[1], and Shuhei Yamaguchi[1]

[1]Departmen of Neurology, Shimane University, Shimane, Japan.
*Correspondence to: onodak1@med.shimane-u.ac.jp (K.O.)

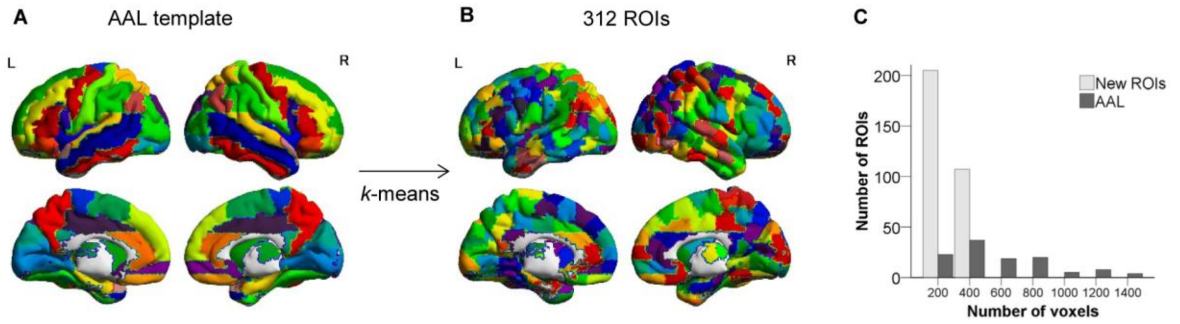

Figure S1. (A) Automated Anatomical Labelling (AAL) template. (B) New region-of-interest (ROI) made by applying k-means method to AAL template. (C) Distribution of region size in AAL and new template.

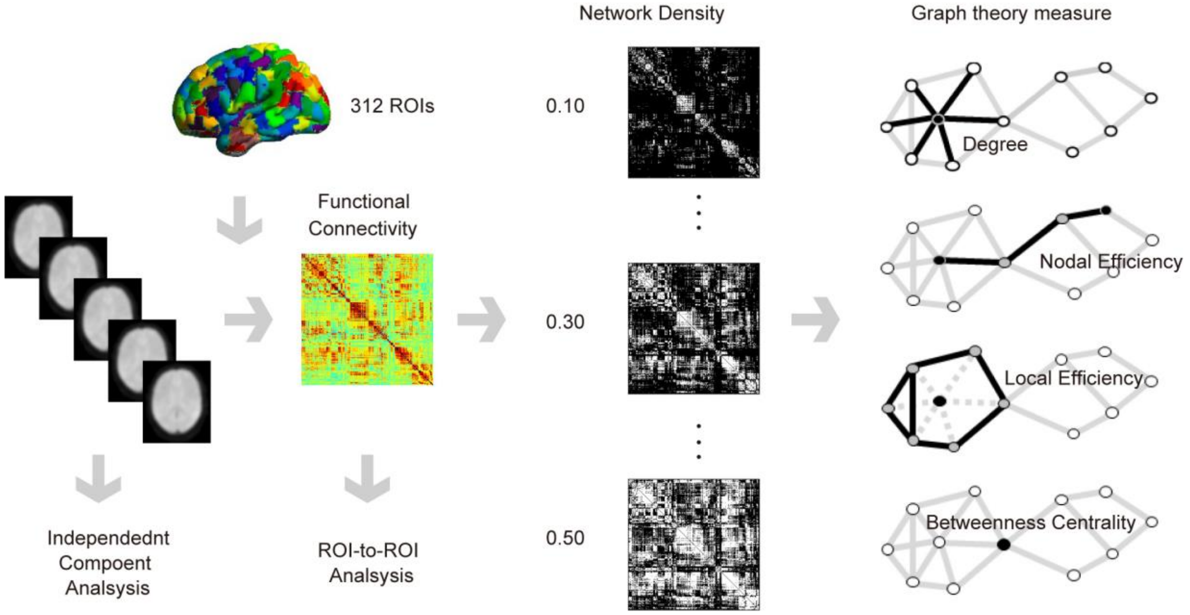

Figure S2. Analysis flow.

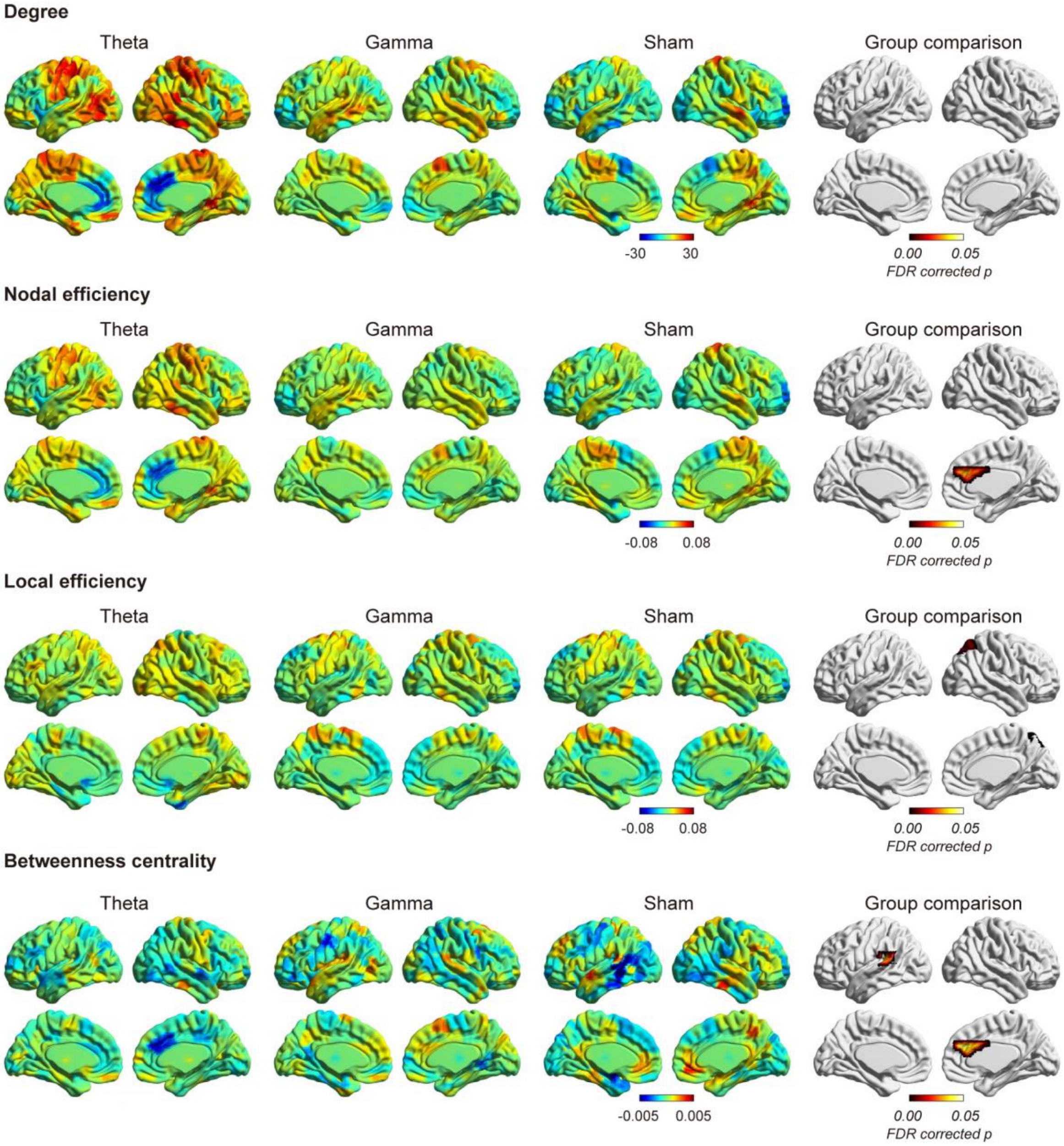

Figure S3. Result of graph theory analysis based on absolute threshold approach. Bilateral and medial views of graph measure changes in theta band, gamma band, and sham tACS groups. Values are represented by average of r threshold range 0.6 - 0.5. Upper limit was selected based on average (0.70 ± 0.08) of connectivity criteria which all nodes have a degree one or more. Hot and cool colors denote increase or decrease of nodal efficiency, respectively.

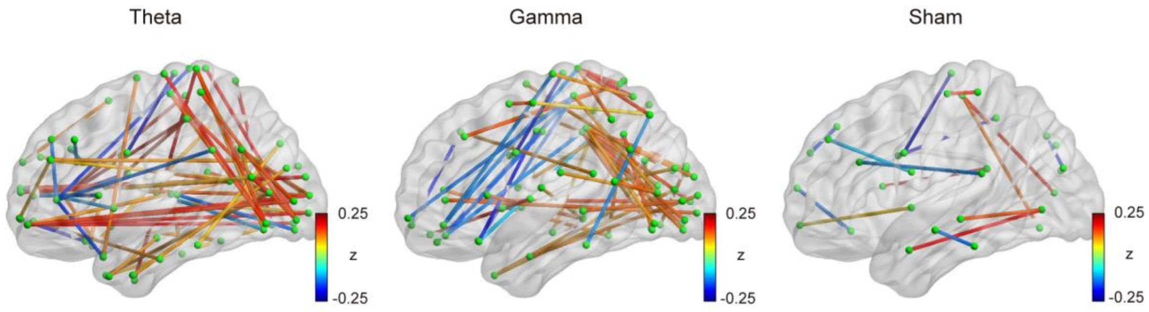

Figure S4. Result of region-of-interest (ROI) based analysis. Each figure shows comparisons of ROI-to-ROI connectivity (z) between pre- and post-tACS in each group. Statistical tests were completed using the Network Based Statistics toolbox and criteria were set at FDR-corrected $p < 0.05$ (Iterations: 5000). Only significant edges were drawn. Hot and cool lines denote increased or decreased functional connectivity by manipulation of tACS, respectively. Group comparison using the same criteria revealed no significant difference between groups.

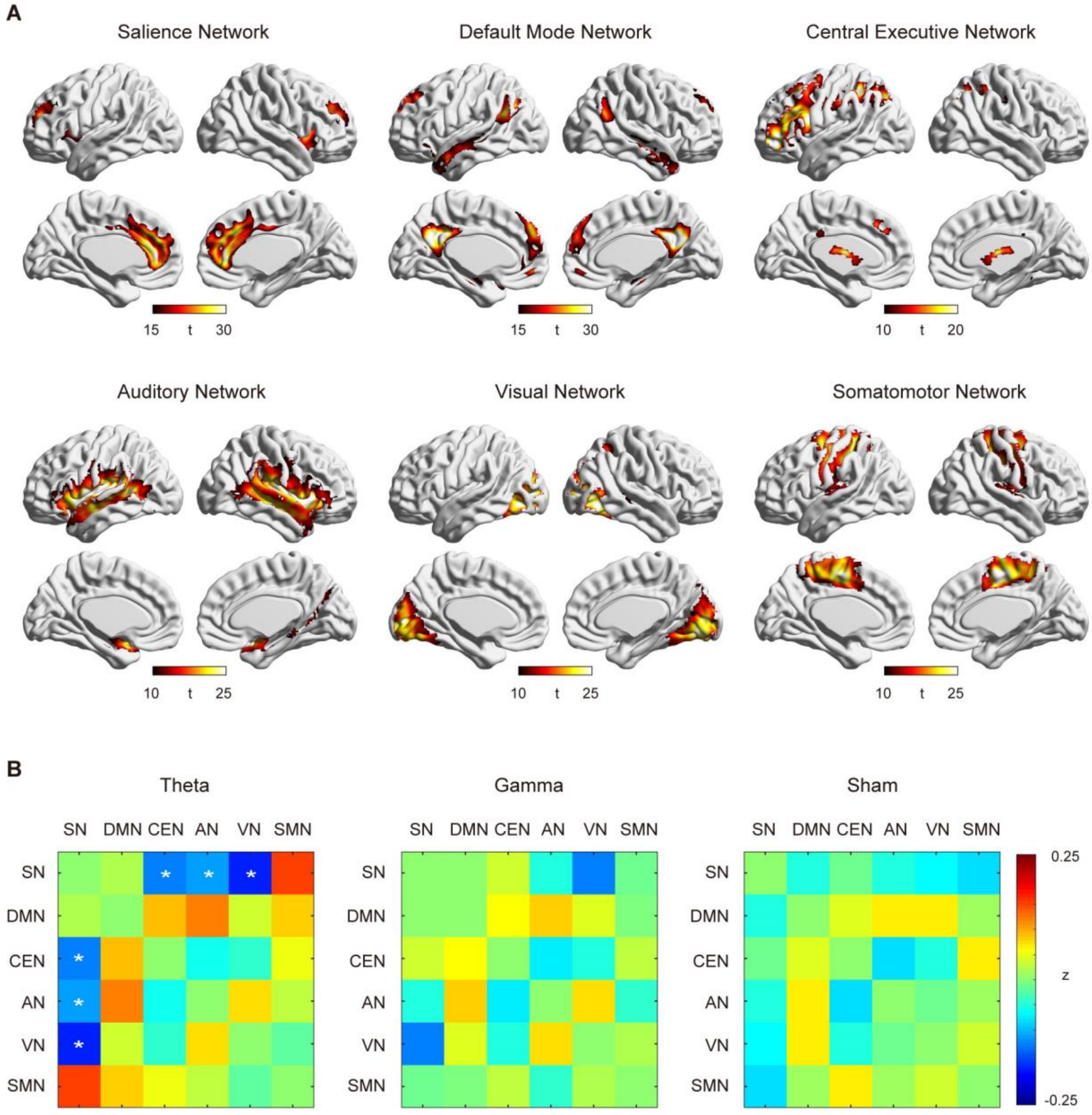

Figure S5. Independent component analysis (ICA). Group ICA fMRI Toolbox (GIFT) was used to detect resting-state networks. Parameters setting in ICA were default values of GIFT. (A) Salience (SN), default mode (DMN), central executive (CEN), auditory (AN), visual (VN), and somatosensory networks (SMN) were separated. Connectivity within each network did not show any effects of tACS. (B) Effects of tACS (post - pre) on connectivity (z) of inter-networks. Hot and cool colors denote increase or decrease functional connectivity between independent networks, respectively. * Denotes significant tACS effect with FDR corrected $p < 0.05$. Group comparison showed no significant differences between groups.

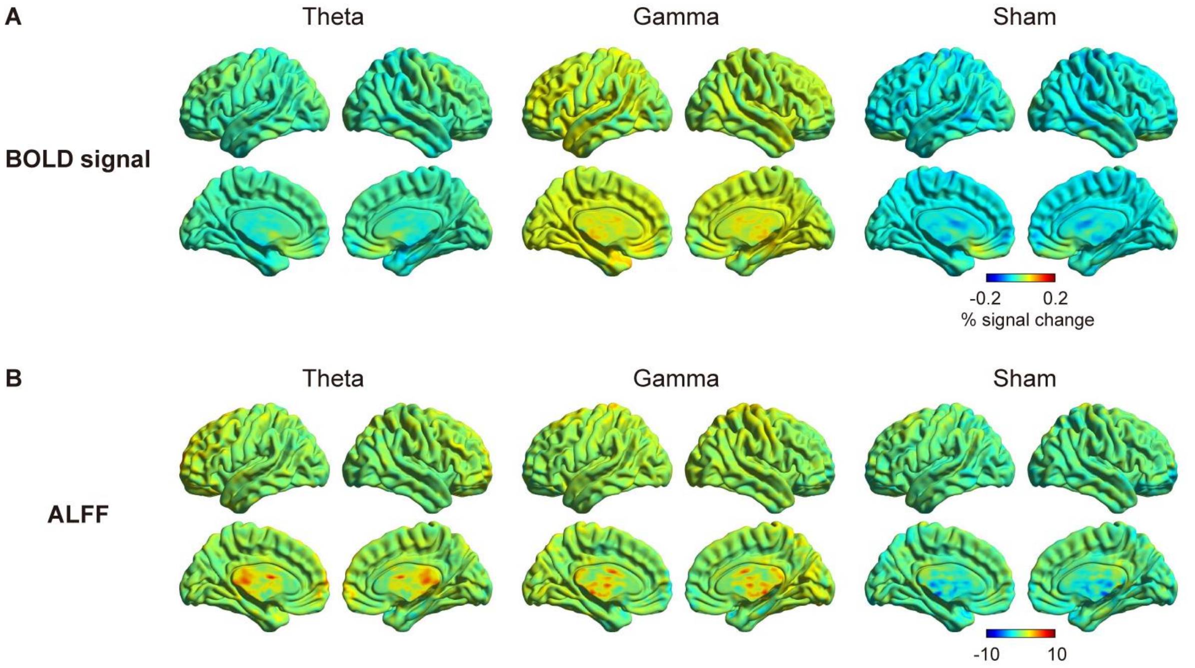

Figure S6. Blood oxygenation level dependent (BOLD) signal and amplitude of low frequency fluctuations (ALFF). (A) BOLD signal was averaged across scans in each individual and each session and % signal changes were calculated as ratios to mean value of pre-tACS session within gray matter at the individual level. Each brain map shows difference of post - pre tACS session. (B)ALFF maps in each group were also shown as the difference of post and pre tACS session. Applying liberal criteria (uncorrected $p < 0.001$ at voxel level, and uncorrected $p < 0.05$ at cluster level), there was no significant group difference of BOLD signal and ALFF between groups.

Supplementary Table S1. Correlation coefficient between tACS-induced changes of graph theoretical measures of right dorsal anterior cingulate cortex and PANAS scores.

	Theta			Gamma			Sham		
	Total	Positive	Negative	Total	Positive	Negative	Total	Positive	Negative
Degree	0.50	0.41	-0.38	-0.30	-0.25	0.26	0.43	0.38	-0.39
Nodal efficiency	**0.56**	0.46	-0.42	-0.22	-0.20	0.17	0.37	0.32	-0.34
Local efficiency	-0.26	-0.15	0.27	-0.17	0.00	0.35	0.19	0.18	-0.16
Betweenness centrality	0.40	0.35	-0.29	-0.06	-0.07	0.03	0.09	0.10	-0.07

PANAS scores were normalized by min-max methods at individual level. Positive and reversed negative scores were summed to total emotional state as total score. Bold value means a significant correlation coefficient ($p<0.05$).

Supplementary Table S2. Correlation coefficient between graph theoretical measures of right dorsal anterior cingulate cortex and PANAS scores at pre- and post-tACS.

	Theta			Gamma			Sham		
	Total	Positive	Negative	Total	Positive	Negative	Total	Positive	Negative
Pre-tACS									
Degree	0.36	0.50	-0.12	-0.23	-0.49	-0.18	-0.12	0.33	0.41
Nodal efficiency	0.38	0.49	-0.15	-0.21	-0.50	-0.21	-0.16	0.32	0.45
Local efficiency	-0.30	-0.31	0.17	-0.17	-0.18	0.01	0.06	0.27	0.13
Betweenness centrality	0.51	0.43	-0.36	0.15	-0.15	-0.26	-0.09	0.21	0.27
Post-tACS									
Degree	0.05	0.10	-0.03	-0.04	0.27	-0.23	-0.06	0.19	-0.19
Nodal efficiency	-0.02	0.20	-0.13	0.05	0.31	-0.16	-0.08	0.20	-0.20
Local efficiency	0.36	-0.13	0.32	-0.20	-0.43	0.09	0.18	0.05	0.07
Betweenness centrality	-0.36	0.00	-0.25	-0.06	0.44	-0.37	-0.20	-0.13	-0.02

PANAS scores were normalized by min-max methods at individual level. Positive and reversed negative scores were summed to total emotional state as total score. No significant relationship was observed in this table.
